# Effects of Foot Progression Angle and Stance Width on Lower-Limb Muscle Activation During the Holding Phase of Forward Lunge Exercises in Healthy Adults: A Randomized Within-Participant Crossover Study

**DOI:** 10.3390/jcm15145567

**Published:** 2026-07-15

**Authors:** Ho-Jin Shin, Hong-Min Kim, Hwi-Young Cho, Sung-Hyeon Kim

**Affiliations:** 1 Wellness Center, Ansan University, Ansan 15328, Republic of Korea; hojin0911@ansan.ac.kr; 2 Kangbuk Samsung Hospital, Yongin 17113, Republic of Korea; khm1541@gmail.com; 3 Department of Physical Therapy, Gachon University, Incheon 21936, Republic of Korea

**Keywords:** surface electromyography, forward lunge exercise, foot progression angle, stance width, lower limb, muscle activation, biomechanics, randomized crossover study

## Abstract

**Background/Objectives:** This study examined whether foot progression angle and stance width modify lower-limb muscle activation during the holding phase of forward lunges in healthy adults. **Methods:** Thirty-six healthy adults completed five randomized within-participant lunge conditions: standard stance with 0°, 30°, and 60° foot progression angles and narrow and wide stances with a 0° foot progression angle. Surface electromyographic activity was recorded from the rectus femoris, gluteus medius, vastus lateralis, vastus medialis, biceps femoris, and semitendinosus. The central 2 s of each 6 s trial were analyzed and normalized to maximal voluntary contraction. Data were analyzed using repeated-measures analysis of variance with Bonferroni-adjusted pairwise comparisons. **Results:** Foot progression angle significantly affected rectus femoris, gluteus medius, vastus lateralis, and vastus medialis activation. Rectus femoris and vastus lateralis activation were higher at 60° than at 0° and 30°, vastus medialis activation was higher at 60° than at 30° only, and gluteus medius activation decreased as foot progression angle increased. Stance width significantly affected rectus femoris, gluteus medius, vastus lateralis, and vastus medialis activation. Rectus femoris activation increased with stance width, gluteus medius activation was lower in standard and wide stances than in narrow stance, and vastus lateralis and vastus medialis activation were higher in standard and wide stances than in narrow stance. Biceps femoris and semitendinosus did not differ across conditions. **Conclusions:** Foot progression angle and stance width modified selected acute lower-limb EMG responses during the holding phase of bodyweight forward lunges.

## 1. Introduction

Lower-limb muscle activation is essential for weight-bearing movement, postural stability, and functional performance [1,2]. Major lower-limb muscle groups, including the quadriceps, hamstrings, and gluteal muscles, contribute to knee control, hip and pelvic stabilization, and movement control during functional and exercise tasks [3,4,5,6]. Because muscle activation patterns can vary according to task posture, understanding how modifiable exercise positions influence lower-limb muscle activation may help clarify the neuromuscular demands of commonly used rehabilitation and training exercises.

Resistance exercises, including squats, deadlifts, and lunges, are widely used in rehabilitation and conditioning programs to improve lower-limb strength, neuromuscular control, and task-specific performance [7,8,9,10,11,12,13,14]. These exercises can impose different neuromuscular demands depending on task configuration, loading condition, and support strategy [7,8,9,10,11]. Because lower-limb strength and muscle function are closely related to gait performance, balance ability, and functional independence, clarifying how exercise posture influences lower-limb muscle activation is relevant for interpreting the functional demands of lower-limb rehabilitation and training exercises [12,13,14].

Forward lunges are commonly used functional exercises for lower-limb strengthening, neuromuscular control, and movement training [15,16,17]. As unilateral weight-bearing tasks, forward lunges require the front limb to support body weight while coordinated control of the knee, hip, pelvis, and trunk is maintained [15,17,18,19,20]. During the lunge movement, the knee extensors contribute to knee extension and control of the flexed-knee position, the hamstrings assist with lower-limb stabilization, and the gluteus medius contributes to pelvic and frontal-plane control [16,19,20]. These characteristics make forward lunges a useful exercise model for examining lower-limb EMG activity under task-specific postural demands. Because forward lunges can be modified by changing step length, foot orientation, stance width, and external load, they are suitable for investigating how modifiable posture variables influence lower-limb muscle activation.

Several studies have examined how modifications in lunge and related closed-chain lower-limb exercise posture influence lower-limb muscle activation and task-related mechanical demands [21,22,23,24,25]. Step length and anterior–posterior foot-position parameters have been reported to influence lower-limb muscle activation during lunge exercises [22,25]. Ankle or foot orientation has also been associated with changes in knee extensor activation during lunge or comparable closed-chain lower-limb tasks [21,24]. In addition, stance width and toe direction have been examined in squat-based tasks, suggesting that mediolateral foot placement may influence lower-limb demands during closed-chain exercise [23]. Collectively, these findings indicate that relatively simple changes in exercise posture can modify lower-limb EMG responses during closed-chain lower-limb exercises.

However, several issues remain insufficiently understood. First, although ankle or foot orientation has been examined in lunge exercises and related lower-limb tasks, direct evidence regarding foot progression angle during forward lunges remains limited [21,24]. Second, although stance width and toe direction have been investigated in squat-based tasks, less is known about how mediolateral stance width affects lower-limb EMG activity during forward lunges [23]. Third, previous lunge studies have primarily focused on step length, sagittal-plane ankle or knee position, selected muscle groups, or mechanical loading, whereas phase-specific EMG activity during the holding phase has received limited attention [22,25]. This distinction is relevant because the holding phase requires participants to maintain body position at approximately 90° of front knee flexion, which may involve neuromuscular demands that differ from those of the descending or ascending phases.

To address these gaps, the present study examined lower-limb EMG activity during the holding phase of forward lunges while modifying foot progression angle and stance width. Foot progression angle was compared under standard stance conditions, whereas stance width was compared with the foot progression angle fixed at 0°. This structure allowed the effects of these two modifiable posture variables to be evaluated separately within the same participants, thereby reducing between-participant variability in EMG responses. We hypothesized that both foot progression angle and stance width would produce muscle-specific changes in lower-limb activation during the holding phase. Accordingly, the present investigation compared lower-limb muscle activation during the forward-lunge holding phase across predefined foot progression angle and stance-width conditions in healthy adults.

## 2. Materials and Methods

### 2.1. Study Design and Participants

This study used a randomized within-participant laboratory crossover design to examine the effects of foot progression angle and stance width on lower-limb muscle activation during the holding phase of forward lunge exercises [26]. Each participant performed all experimental lunge conditions in a randomized order, allowing within-participant comparisons of muscle activation across different posture conditions. Healthy adults residing in Incheon, Republic of Korea, were recruited through social media advertisements and local bulletin board postings between May and August 2024. Ethical approval was granted by the Institutional Review Board of Gachon University on 20 May 2021 (Approval No. 1044396-202104-HR-077-01). The trial was registered with the Clinical Research Information Service of the Republic of Korea on 23 December 2021 (Registration No. KCT0006862). Before enrollment, the study purpose, procedures, and possible risks were explained to each participant, who then provided written informed consent.

The inclusion criteria were as follows [27,28,29]: (1) ability to accurately perform a forward lunge movement, (2) age of 18–40 years, (3) body mass index of 18.5–24.9 kg/m^2^, and (4) no regular exercise participation during the previous 6 months, defined as exercising at least twice per week. The exclusion criteria were as follows [27,28,29]: (1) history of musculoskeletal disorders or previous lower-limb surgery; (2) use of medications that could affect musculoskeletal function; (3) open wounds at the electrode attachment sites; (4) diagnosis of hypertension, hypotension, or other cardiovascular diseases; (5) restricted joint range of motion that could interfere with lunge performance, such as inability to flex the knee to 90° or position the foot at a 60° foot progression angle; and (6) cognitive or mental impairments that could interfere with understanding or performing the study procedures.

A preliminary screening was conducted to confirm each participant’s ability to perform the lunge movement and to assess general physical condition. An a priori sample-size calculation was performed using G*Power version 3.1.9.7 (Heinrich-Heine University Düsseldorf, Düsseldorf, Germany). In the absence of previous research with an identical design, Cohen’s conventional medium effect size (f = 0.25) was assumed [30]. With α = 0.05 and 80% power, 28 participants were required. Allowing for 10% attrition increased the recruitment target to 32 participants; ultimately, 36 participants were enrolled.

### 2.2. Experimental Procedure

**Figure 1:**
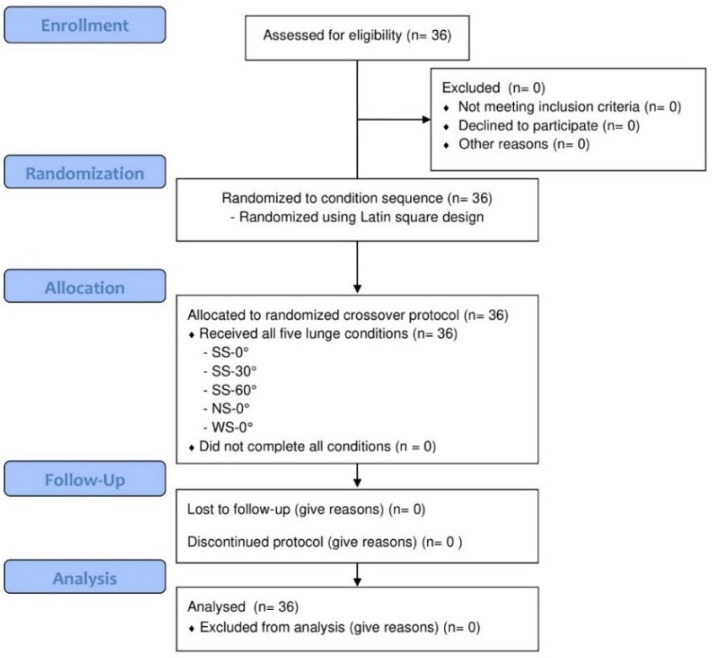
Flow diagram of participant recruitment and experimental procedure.

Figure 1 summarizes the experimental procedure. Before the experiment, participants’ general characteristics were assessed, including height, weight, body mass index, and dominant-side leg length. Participants first completed 5 min of light stretching to prepare for the experimental tasks and reduce injury risk before electrode placement. After stretching, surface electromyography (sEMG) electrodes were placed over the dominant lower-limb muscles. Maximal voluntary contraction (MVC) measurements were obtained in standardized positions for sEMG normalization [31], with at least 1 min between trials. Participants performed forward lunges under five experimental conditions in a randomized order using a Latin square design. The Latin square sequences were computer-generated in advance by a researcher, and each participant was randomly assigned to one of the predefined sequences. The allocation sequence was stored on a restricted-access computer and was disclosed immediately before the experimental testing session. The researcher responsible for participant enrollment and condition-sequence assignment did not have access to the full allocation sequence before assignment. This approach was used to counterbalance the order of experimental conditions across participants. Each condition was performed three times. Recovery periods were set at 1 min between repetitions and 3 min between conditions to limit fatigue accumulation. A licensed physical therapist with over five years of clinical experience supervised all lunge trials for performance accuracy and safety. Participants were monitored for discomfort, pain, or adverse events throughout the experimental procedure. During the experiment, participants used a mirror positioned in front of them to provide visual feedback and assist with postural correction. The examiner provided standardized verbal cues when necessary to maintain movement consistency [32]. Testing took place in a controlled laboratory at Gachon University. The laboratory temperature was maintained at 24 °C, lighting conditions were kept consistent, and the laboratory door was closed during testing to minimize external noise and environmental distractions.

### 2.3. Lunge Exercise Conditions

Participants performed forward lunge exercises under five randomized conditions (Table 1). The dominant leg was determined using at least two of the following three tasks: (1) imagining kicking a ball placed in front of the participant, (2) stepping onto a platform positioned in front of the participant, and (3) stepping on an imaginary object on the ground [33]. All lunge exercises were performed with the dominant leg positioned forward. All lunge trials were performed while participants wore socks without shoes. In this study, foot progression angle was operationally defined as the angle between the heel-to-second-toe line and the forward direction of the lunge.

Step length was standardized for each participant according to the distance from the greater trochanter to the floor to account for individual differences in body size. Participants performed each lunge with both hands placed on their hips. During the descending phase, the front knee was flexed to approximately 90°, and participants were instructed to maintain an upright trunk and avoid excessive medial or lateral knee displacement [25,34]. Participants were instructed not to allow the rear knee to contact the floor during the lunge. A standardized lunge performance guide, including written instructions and visual examples, was provided before testing.

Metronome-paced trials lasted 6 s, with 2 s allocated to descent, 2 s to holding, and 2 s to ascent (60 beats/min). During familiarization, participants practiced the same 6 s movement sequence using the metronome. Side-view video feedback (Samsung digital camera, Samsung Electronics Co., Ltd., Suwon, Republic of Korea) was provided during familiarization to help participants reach approximately 90° of front knee flexion during the holding phase. During testing, the examiner monitored whether participants maintained the instructed movement timing and approximately 90° of front-knee flexion during the holding phase. Participants received standardized verbal cues when necessary, such as “lower slowly” and “keep the knee from collapsing inward,” to maintain consistent movement performance. A trial was repeated if trunk movement was visually estimated to exceed approximately 10°, foot placement deviated by more than 2 cm from the reference line marked on the floor, or the instructed timing or approximate holding posture was not maintained. Trunk position and knee alignment were qualitatively monitored by the examiner using direct observation and side-view video feedback during familiarization and testing. Foot placement was checked using floor markings. Excessive compensatory movements, such as marked trunk lean or pelvic rotation, were corrected using standardized verbal cues when necessary.

**Figure 2:**
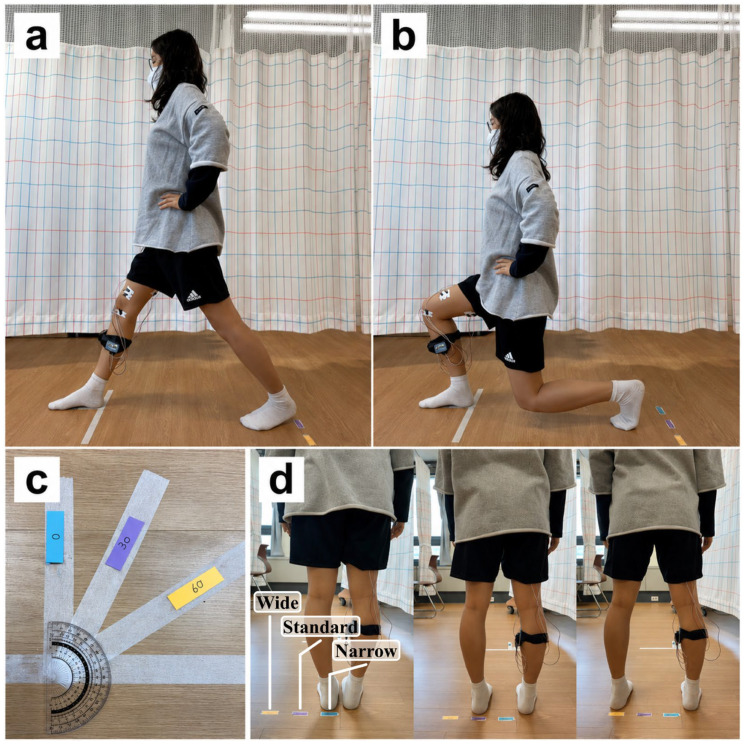
Lunge exercise positions and measurement guides. (**a**) Starting position; (**b**) lunge position at approximately 90° knee flexion; (**c**) foot progression angle guide for 0°, 30°, and 60°; (**d**) narrow, standard, and wide stance-width conditions, defined as one foot width narrower than, equal to, and one foot width wider than pelvic width, respectively.

Foot progression angle was set at 0°, 30°, and 60° using masking tape markers placed on the laboratory floor. The 0°, 30°, and 60° conditions were selected to provide a graded experimental range of foot progression angles under standardized laboratory conditions [21]. Participants aligned the heel-to-second-toe line with the designated angle markers to standardize foot orientation. Stance width was defined relative to each participant’s pelvic width. Standard stance was defined as a stance width equal to pelvic width. Narrow and wide stances were defined as one foot width narrower or wider than pelvic width, respectively, to provide individualized mediolateral stance modifications [23,25]. The lunge positions, foot progression angle guide, and stance-width conditions are shown in Figure 2.

### 2.4. Surface Electromyography Measurement

sEMG was used to assess lower-limb muscle activation during the holding phase of forward lunge exercises [35]. Electromyographic activity was recorded from six muscles of the dominant lower limb: rectus femoris, gluteus medius, vastus lateralis, vastus medialis, biceps femoris, and semitendinosus. These muscles were selected because they are involved in knee extension, hip and pelvic stabilization, knee flexion, and lower-limb control during lunge movements.

A BIOPAC MP160 acquisition system with AcqKnowledge version 5.0 (BIOPAC Systems, Inc., Goleta, CA, USA) was used for sEMG recording and processing. Signals were sampled at 1000 Hz and expressed in millivolts. Before analysis, the EMG signals underwent 30–500 Hz band-pass filtering, full-wave rectification, and root mean square transformation [35]. Before and during data collection, EMG signal quality was checked in real time using AcqKnowledge software. Trials were repeated if electrode detachment, cable movement, signal loss, excessive noise, or obvious motion artifacts were observed. After data collection, raw EMG signals were visually inspected before processing to identify abnormal spikes, signal loss, or excessive noise.

**Figure 3:**
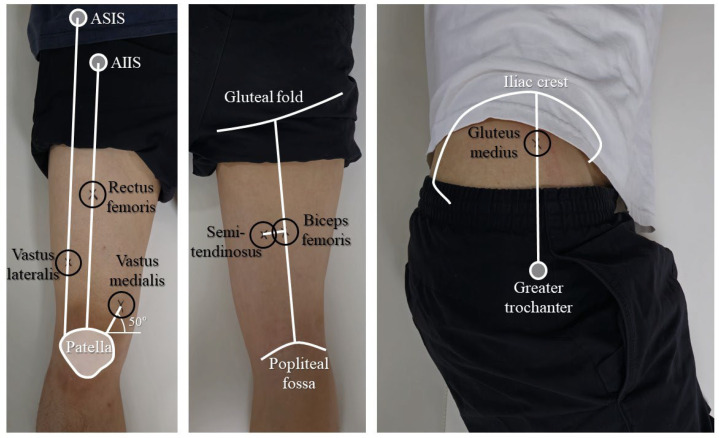
Surface electromyography electrode placement sites for the six recorded lower-limb muscles.

Electrode placement followed the SENIAM recommendations to improve measurement reliability and reproducibility and to reduce potential cross-talk between adjacent muscles [36]. Disposable Ag/AgCl surface electrodes with a 10 mm diameter were used. Hair was removed as needed, and the skin was cleansed with alcohol before electrode application to lower skin impedance. Muscle-specific electrode sites were standardized and are detailed in Table 2 and illustrated in Figure 3.

Muscle activation was normalized to MVC values and expressed as %MVC [31,35]. MVC measurements were performed in standardized testing positions. Quadriceps MVCs for the rectus femoris, vastus lateralis, and vastus medialis were assessed during knee extension initiated at 90° of knee flexion with the foot unsupported. Gluteus medius MVC testing was performed in side-lying position with the upper leg positioned at 20° of abduction and 10° of external rotation. For the biceps femoris and semitendinosus, participants were positioned prone and performed knee flexion. The examiner observed all MVC attempts and used verbal and visual cues to promote maximal and consistent effort; successive trials were separated by at least 1 min. For each muscle, three 5-s MVC trials were obtained. RMS was calculated from the middle 3 s of each trial, and the largest of the three values served as the normalization reference. After completion of the MVC measurements, participants rested for 5 min before performing the forward lunge trials.

During each lunge trial, sEMG activity was recorded for 6 s. The central 2 s of the signal, corresponding to the holding phase at approximately 90° of front knee flexion, were used for analysis to minimize the influence of movement initiation and termination. The descending and ascending phases were not included in the EMG analysis. The final value for each condition was calculated as the mean of three trials.

### 2.5. Statistical Analysis

All analyses were conducted using SPSS version 25.0 (IBM Corp., Armonk, NY, USA). Participant characteristics, including age, height, weight, body mass index, and dominant-side leg length, were summarized descriptively. Continuous data are reported as mean ± standard deviation unless otherwise specified.

Normality of the residuals from each repeated-measures model was evaluated using the Shapiro–Wilk test. Separate one-way repeated-measures analyses of variance were then used to evaluate holding-phase muscle activation according to foot progression angle and stance width. For the foot progression angle analysis, holding-phase muscle activation was compared across the SS-0°, SS-30°, and SS-60° conditions. For the stance width analysis, holding-phase muscle activation was compared across the NS-0°, SS-0°, and WS-0° conditions.

Sphericity was examined using Mauchly’s test, with Greenhouse–Geisser-adjusted results used when this assumption was not satisfied. Significant main effects were followed by Bonferroni-adjusted pairwise comparisons. Mean differences and Bonferroni-adjusted 95% confidence intervals were additionally reported for statistically significant pairwise comparisons. Repeated-measures ANOVA results are reported with F-statistics, degrees of freedom, *p*-values, and partial eta squared (η^2^p) as the effect-size measure. An alpha level of 0.05 was adopted for statistical significance.

## 3. Results

### 3.1. General Characteristics of Participants

A total of 36 healthy adults participated in this study and completed all experimental procedures. No participants withdrew from the study, no adverse events were reported, and no modifications were made to the study protocol during the research period. Participant characteristics are summarized in Table 3. Each enrolled participant satisfied the eligibility criteria.

### 3.2. Statistical Assumption Checks

Shapiro–Wilk tests of model residuals indicated no significant departures from normality for any muscle in either comparison set (all *p* > 0.05). Mauchly’s test indicated violations of sphericity for the biceps femoris in the foot progression angle analysis (W = 0.812, *p* = 0.029) and the rectus femoris in the stance-width analysis (W = 0.612, *p* < 0.001); Greenhouse–Geisser corrections were therefore applied to these two analyses. Sphericity was satisfied for all other muscle-specific analyses (all *p* > 0.05). Mean differences and Bonferroni-adjusted 95% confidence intervals for statistically significant pairwise comparisons are provided in Supplementary Table S1.

### 3.3. Muscle Activation According to Foot Progression Angle

Table 4 presents lower-limb muscle activation across foot progression angle conditions during the holding phase of forward lunge exercises. Significant main effects of foot progression angle were observed for the rectus femoris [F(2,70) = 6.18, *p* = 0.003, η^2^p = 0.150], gluteus medius [F(2,70) = 118.93, *p* < 0.001, η^2^p = 0.773], vastus lateralis [F(2,70) = 7.13, *p* = 0.002, η^2^p = 0.169], and vastus medialis [F(2,70) = 4.03, *p* = 0.022, η^2^p = 0.103]. Pairwise comparisons indicated greater rectus femoris activation in SS-60° than in both SS-0° and SS-30°. Gluteus medius activation decreased as foot progression angle increased, with significant differences among all three conditions. Vastus lateralis activation was higher in the SS-60° condition than in the SS-0° and SS-30° conditions. Vastus medialis activation was higher in SS-60° than in SS-30°, whereas SS-0° and SS-60° did not differ significantly. No significant main effects were observed for biceps femoris or semitendinosus activation across the foot progression angle conditions.

### 3.4. Muscle Activation According to Stance Width

Table 5 presents lower-limb muscle activation across stance-width conditions during the holding phase of forward lunge exercises. Significant main effects of stance width were observed for the rectus femoris [F(1.44,50.45) = 58.33, *p* < 0.001, η^2^p = 0.625], gluteus medius [F(2,70) = 9.60, *p* < 0.001, η^2^p = 0.215], vastus lateralis [F(2,70) = 9.80, *p* < 0.001, η^2^p = 0.219], and vastus medialis [F(2,70) = 10.79, *p* < 0.001, η^2^p = 0.236]. Rectus femoris activation increased stepwise across NS-0°, SS-0°, and WS-0°, with significant differences in all pairwise comparisons. Gluteus medius activation was lower in the SS-0° and WS-0° conditions than in the NS-0° condition, whereas the difference between SS-0° and WS-0° was not significant. Vastus lateralis and vastus medialis activation were higher in the SS-0° and WS-0° conditions than in the NS-0° condition, whereas the differences between SS-0° and WS-0° were not significant. No significant main effects were observed for biceps femoris or semitendinosus activation across the stance width conditions.

## 4. Discussion

In this within-participant study, lower-limb EMG responses during the holding phase were evaluated across foot progression angle and stance-width conditions in healthy adults performing forward lunges. The main findings were that a 60° foot progression angle was associated with higher rectus femoris and vastus lateralis activation than the 0° and 30° conditions, whereas vastus medialis showed only a condition-specific increase and gluteus medius activation decreased as foot progression angle increased. Stance width also influenced selected lower-limb muscle activation, but the response pattern differed across muscles: rectus femoris activation increased as stance width increased, whereas vastus lateralis and vastus medialis activation were higher and gluteus medius activation was lower in the standard and wide stance conditions than in the narrow stance condition. In contrast, biceps femoris and semitendinosus activation did not differ significantly according to foot progression angle or stance width. Overall, these findings indicate that foot progression angle and stance width modified selected acute lower-limb EMG responses during the holding phase of forward lunges in healthy adults.

Foot progression angle produced muscle-specific responses during the holding phase of forward lunges. In the present study, rectus femoris and vastus lateralis activation were higher in the 60° condition than in the 0° and 30° conditions, whereas vastus medialis showed a less consistent, condition-specific increase. These findings suggest that the larger-angle condition may be associated with altered knee extensor demand during the holding phase, possibly by changing the orientation of the foot and lower limb relative to the forward lunge direction. This interpretation is consistent with previous findings showing that foot orientation, ankle joint position, or foot position can influence knee extensor or quadriceps activation during lunge exercises, lower-limb exercises, and functional weight-bearing tasks [21,37,38,39]. In contrast, gluteus medius activation decreased progressively as foot progression angle increased. Because the gluteus medius contributes to hip and pelvic control during lower-limb tasks [40], this decrease may reflect a change in hip or pelvic control strategy associated with the altered foot orientation. Taken together, the responses to foot progression angle were not uniform across muscles: gluteus medius activation showed a consistent graded decrease across the tested angles, whereas the quadriceps responses were less consistent and were largely confined to the 60° condition. This distinction suggests that foot progression angle was associated with qualitatively different, muscle-specific EMG response patterns rather than a general increase or decrease in lower-limb activation during the holding phase of forward lunges.

Stance width also affected knee extensor activation during the holding phase of forward lunges, but the response pattern differed among the knee extensor muscles. Rectus femoris activation increased progressively from the narrow to standard and wide stance conditions, suggesting that this muscle was particularly responsive to changes in mediolateral stance width. In contrast, vastus lateralis and vastus medialis activation were higher in the standard and wide stance conditions than in the narrow stance condition, but did not differ significantly between the standard and wide stance conditions. This pattern suggests that the narrow stance condition was associated with lower activation of the vasti muscles relative to the standard and wide stance conditions, whereas increasing stance width beyond the standard stance did not produce additional increases in vastus lateralis or vastus medialis activation. One possible explanation is that changing mediolateral stance width altered task configuration and front-limb positioning during the holding phase, which may have influenced knee extensor demand without affecting all knee extensor muscles in the same manner. This interpretation is broadly consistent with previous studies showing that stance width, toe direction, or foot-position parameters can modify lower-limb demands or muscle activation during closed-chain lower-limb exercises and lunge tasks [22,23,25]. Thus, the knee-extensor response to stance width was not uniform: rectus femoris showed a graded increase across the tested stance widths, whereas the vasti showed a more condition-dependent pattern characterized by lower activation in the narrow stance and no additional increase from the standard to the wide stance. This divergence suggests differential responsiveness of the knee extensor muscles to mediolateral stance modification.

In contrast to the knee extensor pattern, stance width produced a distinct response in the gluteus medius. Gluteus medius activation was higher in the narrow stance condition than in the standard and wide stance conditions, whereas the standard and wide stance conditions did not differ significantly from each other. This pattern should therefore be interpreted as a narrow-stance-related increase in gluteus medius activation rather than as a progressive decrease in gluteus medius activation with increasing stance width. The gluteus medius contributes to hip and pelvic control, particularly during tasks that require frontal-plane stabilization [40]. One possible explanation is that the narrow stance condition may have reduced mediolateral support during the holding phase, thereby increasing the need for hip or pelvic control strategies involving the gluteus medius. Alternative factors, including hip position, trunk strategy, motor-control variability, perceived stability, or altered weight distribution, may also have contributed to the observed gluteus medius pattern. This interpretation is broadly consistent with previous findings showing that lunge variations with different mediolateral foot positions can influence mediolateral balance and lower-limb muscle activation [41]. However, because postural-control and biomechanical variables were not directly measured, this explanation should be regarded as a plausible interpretation rather than a demonstrated mechanism.

No significant between-condition differences in biceps femoris or semitendinosus activation were observed across either foot progression angle or stance-width conditions during the holding phase. This null finding should not be interpreted as indicating that the hamstrings were not involved in the forward lunge task. The biceps femoris and semitendinosus contribute to knee flexion, hip extension, and lower-limb stabilization, but their activation may be influenced by task phase, loading condition, and movement demands [42]. Under the present bodyweight holding-phase protocol, changes in foot progression angle and stance width may not have provided sufficient additional demand to produce statistically detectable changes in front-limb hamstring EMG activity. Different activation patterns may emerge during dynamic phases or externally loaded lunge variations, which were not examined here. Therefore, the absence of significant biceps femoris and semitendinosus differences supports the interpretation that the posture-related EMG responses observed in this study were muscle-specific and did not extend to all measured lower-limb muscles.

The present findings may provide preliminary information for clinicians, trainers, and researchers who analyze forward lunge variations. Importantly, the practical interpretation of these findings should consider the consistency and absolute magnitude of the EMG response rather than statistical significance alone; isolated condition-specific differences should be interpreted more cautiously than graded patterns observed across conditions. Specifically, foot progression angle and stance width may be considered modifiable posture variables associated with selected acute lower-limb EMG responses during the holding phase of bodyweight forward lunges. Because forward lunges are commonly used in rehabilitation and lower-limb strengthening contexts, understanding how posture-related variables influence acute EMG responses may be useful when interpreting exercise demands or designing future EMG-based exercise studies [42]. However, these findings should be interpreted in the context of healthy adults who did not regularly exercise and performed bodyweight lunges under the present holding-phase protocol. Differences in training status, task familiarity, and clinical characteristics may alter neuromuscular responses to the same postural modification, limiting direct extrapolation of the observed EMG patterns to trained or clinical populations. Therefore, the present results should not be regarded as direct evidence for clinical effectiveness, selective muscle strengthening, balance improvement, or long-term training adaptation.

Several limitations should be noted. The sample size was based on an assumed medium effect and may have been underpowered for smaller muscle-specific effects. Participants were healthy young adults who did not regularly exercise, limiting generalizability to trained, clinical, or sport-specific populations. Foot progression angle and stance width were tested in separate condition sets, precluding interaction analysis. The protocol assessed only front-limb surface EMG during the holding phase of bodyweight forward lunges; therefore, findings may not apply to the rear limb, dynamic phases, or loaded variations. Step length was standardized using greater-trochanter-to-floor distance as an exploratory body-size reference, which may limit comparability with studies using other step-length standardization methods. Because kinematic and kinetic variables, center-of-pressure measures, weight distribution, balance, and functional outcomes were not assessed, biomechanical and postural-control explanations remain inferential. Movement control relied partly on examiner monitoring and video-assisted visual inspection, and MVC normalization postures did not fully replicate lunge joint configurations. Residual fatigue or learning effects cannot be excluded. Further research using larger factorial designs should extend sampling to broader populations and integrate EMG with biomechanical, balance, and functional outcomes.

## 5. Conclusions

This study showed that foot progression angle and stance width influenced selected lower-limb muscle activation during the holding phase of forward lunges in healthy adults. A greater foot progression angle was associated with higher rectus femoris and vastus lateralis activation, a condition-specific increase in vastus medialis activation, and lower gluteus medius activation. Stance width produced muscle-specific responses in the knee extensors and gluteus medius, whereas biceps femoris and semitendinosus did not differ across conditions. These findings indicate that foot progression angle and stance width were each associated with acute lower-limb EMG responses during the holding phase under the specific lunge conditions examined in this study. Future studies should examine dynamic phases and loaded conditions, include clinical or sport-specific populations, and incorporate additional biomechanical outcomes.

## Figures and Tables

**Table 1:** Description of lunge exercise conditions.

Condition	Definition
SS-0°	Standard stance with a 0° foot progression angle
NS-0°	Narrow stance with a 0° foot progression angle
WS-0°	Wide stance with a 0° foot progression angle
SS-30°	Standard stance with a 30° foot progression angle
SS-60°	Standard stance with a 60° foot progression angle

**Table 2:** Electrode placement for electromyography measurements.

Muscle	Electrode Placement
Rectus femoris	At the midpoint of the anterior thigh along the line connecting the anterior inferior iliac spine and proximal patellar border
Gluteus medius	At one-third of the distance from the highest point of the iliac crest toward the greater trochanter
Vastus lateralis	Along the line toward the anterior superior iliac spine, 10 cm proximal to the superolateral patellar border
Vastus medialis	At a site 4 cm proximal to the superomedial patellar border, with an orientation of approximately 50°
Biceps femoris	On the posterior thigh, halfway between the gluteal fold and the popliteal fossa
Semitendinosus	3 cm medial to the biceps femoris electrode placement site

**Table 3:** General characteristics of the study participants.

Variable	Total Participants (*n* = 36)
Sex (male/female)	17/19
Age (years)	28.39 ± 3.07
Height (cm)	165.00 ± 8.33
Weight (kg)	61.94 ± 14.98
Body mass index (kg/m^2^)	22.75 ± 1.85
Dominant-side leg length (cm)	77.11 ± 3.31

Data are presented as the number of participants or as mean ± standard deviation, as appropriate.

**Table 4:** Lower-limb muscle activation according to foot progression angle.

Muscle	SS-0°	SS-30°	SS-60°	F(df)	ANOVA	η^2^p	0–30°	0–60°	30–60°
RF	18.99 ± 6.80	19.09 ± 7.12	20.35 ± 8.01	6.18 (2,70)	0.003	0.150	1.000	0.003	0.026
GM	12.00 ± 3.01	10.01 ± 2.33	7.66 ± 2.30	118.93 (2,70)	<0.001	0.773	<0.001	<0.001	<0.001
VL	35.63 ± 12.55	35.43 ± 10.50	37.54 ± 11.43	7.13 (2,70)	0.002	0.169	1.000	0.022	0.001
VM	36.09 ± 15.99	34.78 ± 14.19	37.50 ± 15.99	4.03 (2,70)	0.022	0.103	0.484	0.506	0.022
BF	4.91 ± 1.56	4.61 ± 2.38	4.91 ± 1.33	0.33 (1.68,58.93)	0.682	0.009	1.000	1.000	1.000
ST	8.98 ± 3.17	8.68 ± 2.36	9.61 ± 2.77	0.98 (2,70)	0.382	0.027	1.000	1.000	0.418

Data are presented as mean ± standard deviation and expressed as %MVC. All analyses included 36 participants. The ANOVA column presents *p*-values for the main effect of foot progression angle. Pairwise comparison *p*-values were adjusted using the Bonferroni correction. Greenhouse–Geisser-adjusted degrees of freedom are reported for BF because the assumption of sphericity was violated. η^2^p, partial eta squared; BF, biceps femoris; GM, gluteus medius; RF, rectus femoris; ST, semitendinosus; VL, vastus lateralis; VM, vastus medialis; SS-0°, standard stance with a 0° foot progression angle; SS-30°, standard stance with a 30° foot progression angle; SS-60°, standard stance with a 60° foot progression angle.

**Table 5:** Lower-limb muscle activation according to stance width.

Muscle	NS-0°	SS-0°	WS-0°	F(df)	ANOVA	η^2^p	NS–SS	NS–WS	SS–WS
RF	17.36 ± 5.69	18.99 ± 6.80	23.05 ± 9.09	58.33 (1.44,50.45)	<0.001	0.625	<0.001	<0.001	<0.001
GM	13.23 ± 3.54	12.00 ± 3.01	11.80 ± 3.60	9.60 (2,70)	<0.001	0.215	0.004	<0.001	1.000
VL	33.16 ± 9.71	35.63 ± 12.55	36.44 ± 13.25	9.80 (2,70)	<0.001	0.219	0.009	0.001	0.767
VM	32.59 ± 12.52	36.09 ± 15.99	37.20 ± 16.71	10.79 (2,70)	<0.001	0.236	0.005	<0.001	0.800
BF	5.06 ± 1.32	4.91 ± 1.56	5.62 ± 2.43	1.58 (2,70)	0.214	0.043	1.000	0.720	0.369
ST	9.15 ± 3.17	8.98 ± 3.17	8.80 ± 2.63	0.14 (2,70)	0.872	0.004	1.000	1.000	1.000

Data are presented as mean ± standard deviation and expressed as %MVC. All analyses included 36 participants. The ANOVA column presents *p*-values for the main effect of stance width. Pairwise comparison *p*-values were adjusted using the Bonferroni correction. Greenhouse–Geisser-adjusted degrees of freedom are reported for RF because the assumption of sphericity was violated. η^2^p, partial eta squared; BF, biceps femoris; GM, gluteus medius; RF, rectus femoris; ST, semitendinosus; VL, vastus lateralis; VM, vastus medialis; NS-0°, narrow stance with a 0° foot progression angle; SS-0°, standard stance with a 0° foot progression angle; WS-0°, wide stance with a 0° foot progression angle.

## Data Availability

The data that support the findings of this study are available from the corresponding author upon reasonable request. The data are not publicly available due to institutional ethical restrictions and participant confidentiality.

## Supplementary Materials

The following supporting information can be downloaded at: https://www.mdpi.com/article/10.3390/jcm15145567/s1, Table S1: Mean differences and Bonferroni-adjusted 95% confidence intervals for statistically significant pairwise comparisons.

## Abbreviations

The following abbreviations are used in this manuscript:

ANOVA	Analysis of variance
BF	Biceps femoris
GM	Gluteus medius
MSIT	Ministry of Science and ICT
MVC	Maximal voluntary contraction
NRF	National Research Foundation of Korea
NS-0°	Narrow stance with a 0° foot progression angle
RF	Rectus femoris
sEMG	Surface electromyography
SENIAM	Surface Electromyography for the Non-Invasive Assessment of Muscles
SS-0°	Standard stance with a 0° foot progression angle
SS-30°	Standard stance with a 30° foot progression angle
SS-60°	Standard stance with a 60° foot progression angle
ST	Semitendinosus
VL	Vastus lateralis
VM	Vastus medialis
WS-0°	Wide stance with a 0° foot progression angle
η^2^p	Partial eta squared
