# Supplementary material for: Effects of Foot Progression Angle and Stance Width on Lower-Limb Muscle Activation During the Holding Phase of Forward Lunge Exercises in Healthy Adults: A Randomized Within-Participant Crossover Study
Source: J Clin Med. 2026 Jul 15;15(14):5567. doi: 10.3390/jcm15145567 (PMC13413211; doi:10.3390/jcm15145567)
Supplement: Supplementary file 1 [file jcm-15-05567-s001.zip › jcm-4408593-supplementary.pdf]

Table S1. Mean differences and Bonferroni-adjusted 95% confidence intervals for statistically significant pairwise comparisons

**Analysis**	**Muscle**	**Comparison**	**Mean difference (%MVC)**	**Bonferroni-adjusted 95% CI**	**Adjusted p**
Foot progression angle	RF	SS-0° − SS-60°	−1.36	−2.31 to −0.41	0.003
Foot progression angle	RF	SS-30° − SS-60°	−1.26	−2.40 to −0.12	0.026
Foot progression angle	GM	SS-0° − SS-30°	1.99	1.27 to 2.71	<0.001
Foot progression angle	GM	SS-0° − SS-60°	4.34	3.57 to 5.12	<0.001
Foot progression angle	GM	SS-30° − SS-60°	2.35	1.73 to 2.97	<0.001
Foot progression angle	VL	SS-0° − SS-60°	−1.91	−3.60 to −0.22	0.022
Foot progression angle	VL	SS-30° − SS-60°	−2.11	−3.46 to −0.76	0.001
Foot progression angle	VM	SS-30° − SS-60°	−2.72	−5.12 to −0.32	0.022
Stance width	RF	NS-0° − SS-0°	−1.63	−2.54 to −0.72	<0.001
Stance width	RF	NS-0° − WS-0°	−5.69	−7.37 to −4.01	<0.001
Stance width	RF	SS-0° − WS-0°	−4.06	−5.45 to −2.67	<0.001
Stance width	GM	NS-0° − SS-0°	1.23	0.36 to 2.11	0.004
Stance width	GM	NS-0° − WS-0°	1.43	0.54 to 2.32	<0.001
Stance width	VL	NS-0° − SS-0°	−2.47	−4.42 to −0.52	0.009
Stance width	VL	NS-0° − WS-0°	−3.28	−5.38 to −1.18	0.001
Stance width	VM	NS-0° − SS-0°	−3.50	−6.09 to −0.91	0.005
Stance width	VM	NS-0° − WS-0°	−4.61	−7.35 to −1.87	<0.001

Mean difference was calculated as the first-listed condition minus the second-listed condition. Confidence intervals and p-values were adjusted using the Bonferroni correction within each muscle-specific pairwise-comparison family. GM, gluteus medius; NS-0°, narrow stance with a 0° foot progression angle; RF, rectus femoris; SS-0°, standard stance with a 0° foot progression angle; SS-30°, standard stance with a 30° foot progression angle; SS-60°, standard stance with a 60° foot progression angle; VL, vastus lateralis; VM, vastus medialis; WS-0°, wide stance with a 0° foot progression angle.
